# Culturing of ‘unculturable’ human microbiota reveals novel taxa and extensive sporulation

**DOI:** 10.1038/nature17645

**Published:** 2016-05-04

**Authors:** Hilary P. Browne, Samuel C. Forster, Blessing O. Anonye, Nitin Kumar, B. Anne Neville, Mark D. Stares, David Goulding, Trevor D. Lawley

**Affiliations:** 1 Host-Microbiota Interactions Laboratory, Wellcome Trust Sanger Institute, Hinxton, CB10 1SA UK; 2 Centre for Innate Immunity and Infectious Diseases, Hudson Institute of Medical Research, Clayton, 3168 Victoria Australia; 3 Department of Molecular and Translational Sciences, Monash University, Clayton, 3800 Victoria Australia; 4 Microbial Pathogenesis Laboratory, Wellcome Trust Sanger Institute, Hinxton, CB10 1SA UK

**Keywords:** Bacterial genes, Microbiome, Metagenomics, Microbial ecology

## Abstract

Our intestinal microbiota harbours a diverse bacterial community required for our health, sustenance and wellbeing^1,2^. Intestinal colonization begins at birth and climaxes with the acquisition of two dominant groups of strict anaerobic bacteria belonging to the Firmicutes and Bacteroidetes phyla^2^. Culture-independent, genomic approaches have transformed our understanding of the role of the human microbiome in health and many diseases^1^. However, owing to the prevailing perception that our indigenous bacteria are largely recalcitrant to culture, many of their functions and phenotypes remain unknown^3^. Here we describe a novel workflow based on targeted phenotypic culturing linked to large-scale whole-genome sequencing, phylogenetic analysis and computational modelling that demonstrates that a substantial proportion of the intestinal bacteria are culturable. Applying this approach to healthy individuals, we isolated 137 bacterial species from characterized and candidate novel families, genera and species that were archived as pure cultures. Whole-genome and metagenomic sequencing, combined with computational and phenotypic analysis, suggests that at least 50–60% of the bacterial genera from the intestinal microbiota of a healthy individual produce resilient spores, specialized for host-to-host transmission. Our approach unlocks the human intestinal microbiota for phenotypic analysis and reveals how a marked proportion of oxygen-sensitive intestinal bacteria can be transmitted between individuals, affecting microbiota heritability.

**Supplementary information:**

The online version of this article (doi:10.1038/nature17645) contains supplementary material, which is available to authorized users.

## Abstract (web summary)

A novel approach is used to cultivate a substantial proportion of the human gut microbiota, representing an important step forward in characterizing the role of these bacteria in health and disease.

**Supplementary information:**

The online version of this article (doi:10.1038/nature17645) contains supplementary material, which is available to authorized users.

## Metagenomic analysis of human gut microbiota

The human intestinal microbiota comprises perhaps hundreds of bacterial species. Most of them are regarded as 'unculturable' and have never been isolated in the laboratory. Trevor Lawley and colleagues have used a novel approach based on targeted phenotypic culturing linked to large-scale whole-genome sequencing, phylogenetic analysis and computational modelling, to cultivate a substantial proportion of the human gut microbiota. Applying this workflow to faecal samples from healthy individuals, the authors isolated 137 distinct bacterial species as pure cultures, including 45 candidate novel species. More than half of the bacterial genera identified produce resilient spores, specialized for host-to-host transmission. This work demonstrates the value of phenotypic-based studies in determining the role of the gut microbiota in health and disease.

**Supplementary information:**

The online version of this article (doi:10.1038/nature17645) contains supplementary material, which is available to authorized users.

## Main

A typical human intestinal microbiota contains 100–1,000 bacterial species with tremendous compositional diversity between individuals, such that each individual’s microbiota is as unique as a fingerprint^1,4^. Despite the taxonomic diversity, metagenomic sequencing has highlighted that a health-associated intestinal microbiome codes for highly conserved gene families and pathways associated with basic bacterial physiology and growth^2^. However, many basic microbiota functions related to homeostasis, immune system development, digestion, pathogen resistance and microbiota inheritance have yet to be discovered^5^. This formidable challenge to validate and decipher the functional attributes of the microbiota has been hindered because the majority of intestinal bacteria are widely considered to be ‘unculturable’ and have never been isolated in the laboratory^3,6^.

**Figure 1:**
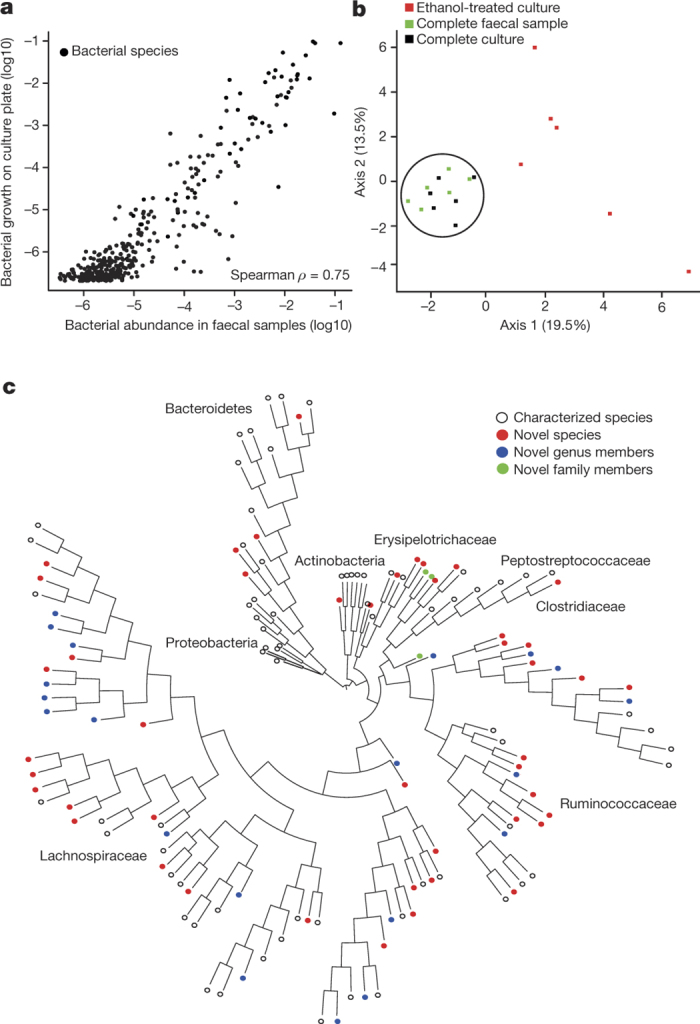
Targeted phenotypic culturing facilitates bacterial discovery from healthy human faecal microbiota. **a**, Relative abundance of bacteria in faecal samples (*x* axis) compared with relative abundance of bacteria growing on YCFA agar plates (*y* axis) as determined by metagenomic sequencing. Bacteria grown on YCFA agar are representative of the complete faecal samples as indicated by Spearman *ρ* = 0.75 (*n* = 6). **b**, Principal component analysis plot of 16S rRNA gene sequences detected from six donor faecal samples (*n* = 6), representing bacteria in complete faecal samples (green), faecal bacterial colonies recovered from YCFA agar plates without ethanol pre-treatment (black) or with ethanol pre-treatment to select for ethanol-resistant spore-forming bacteria (red). Culturing without ethanol selection is representative of the complete faecal sample, ethanol treatment shifts the profile, enriching for ethanol-resistant spore-forming bacteria and allowing their subsequent isolation. **c**, Phylogenetic tree of bacteria cultured from the six donors constructed from full-length 16S rRNA gene sequences. Novel candidate species (red), genera (blue) and families (green) are shown by dot colours. Major phyla and family names are indicated. Proteobacteria were not cultured, but are included for context. PowerPoint slide

**Extended Data Figure 1:**
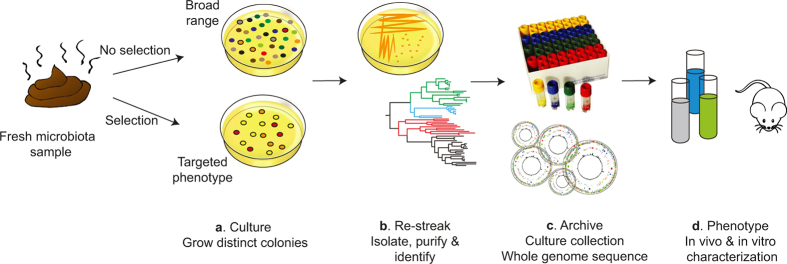
A workflow for culturing, archiving and characterization of the intestinal microbiota. **a**–**d**, Schematic diagram of the workflow, encompassing bacterial culturing and genomics to isolate and characterize bacterial species from the human intestinal microbiota. The process incorporates several steps, which are culture, re-streak, archive and phenotype. **a**, Fresh faecal samples are left untreated or are treated to select for bacteria with a desired phenotype (such as sporulation). The stool is homogenized and then serially diluted and then aliquots of the homogenate are inoculated on YCFA agar to culture bacteria. **b**, Isolates are identified by selecting single colonies that are streaked to purity and full-length 16S rRNA genes are amplified and sequenced. **c**, Each unique, novel and desired isolate is archived frozen in a culture collection and a whole-genome sequence is generated for each. **d**, Phenotypic characterization and functional validation of metagenomics studies can be performed *in vitro* and *in vivo*.

**Extended Data Figure 2:**
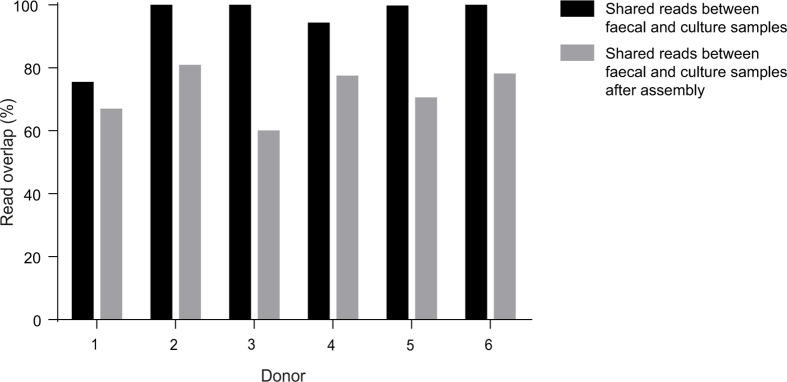
Comparison of sequence read content of faecal samples and cultured samples for six donors. The majority of sequence reads from the original donor faecal samples (*n* = 6) are present in culture samples both as raw reads (93% shared on average across the six donors) and after *de novo* assembly (72% shared on average across the six donors).

We sought to establish a genomic-based workflow that could be used as a platform for targeted culturing of specific bacterial phenotypes (Extended Data Fig. 1). Accordingly, we collected fresh faecal samples from six healthy humans and defined the resident bacterial communities with a combined metagenomic sequencing and bacterial culturing approach. Applying shotgun metagenomic sequencing, we profiled and compared the bacterial species present in the original faecal samples to those that grew as distinct colonies on agar plates containing the complex, broad-range bacteriological medium, YCFA^7^. Importantly, we observed a strong correlation between the two samples at the species level (Spearman’s *ρ* = 0.75, *P* < 0.01) (Fig. 1a). When sequenced, the original faecal sample and the cultured bacterial community shared an average of 93% of raw reads across the six donors. This overlap was 72% after *de novo* assembly (Extended Data Fig. 2). Comparison to a comprehensive gene catalogue that was derived by culture-independent means from the intestinal microbiota of 318 individuals^4^ found that 39.4% of the genes in the larger database were represented in our cohort and 73.5% of the 741 computationally derived metagenomic species identified through this analysis were also detectable in the cultured samples.

Together, these results demonstrate that a considerable proportion of the bacteria within the faecal microbiota can be cultured with a single growth medium. However, more than 8 × 10^6^ distinct colonies would need to be picked from YCFA agar plates to match the species detection sensitivity of metagenomic sequencing. Thus, we established a broad-range culturing method that, when combined with high-throughput archiving or specific phenotypic selection, can be used to isolate and identify novel bacteria from the gastrointestinal tract.

The human intestinal microbiota is dominated by strict anaerobic bacteria that are extremely sensitive to ambient oxygen, so it is not known how these bacteria survive environmental exposure to be transmitted between individuals. Certain members of the Firmicutes phylum, including the diarrhoeal pathogen *Clostridium difficile*, produce metabolically dormant and highly resistant spores during colonization that facilitate both persistence within the host and environmental transmission^8,9,10^. Relatively few intestinal spore-forming bacteria have been cultured to date, and while metagenomic studies suggest that other unexpected members of the intestinal microbiota possess potential sporulation genes, these bacteria remain poorly characterized^11,12,13,14^.

We hypothesized that sporulation is an unappreciated basic phenotype of the human intestinal microbiota that may have a profound impact on microbiota persistence and spread between humans. Spores from *C. difficile* are resistant to ethanol and this phenotype can be used to select for spores from a mixed population of spores and ethanol-sensitive vegetative cells^15^. Faecal samples with or without ethanol treatment were processed using our combined culture and metagenomics workflow (Extended Data Fig. 1). Principle component analysis demonstrated that ethanol treatment profoundly altered the culturable bacterial composition and, when compared to the original profile, efficiently enriched for ethanol-resistant bacteria, facilitating their isolation (Fig. 1b). We picked ~2,000 individual bacterial colonies from both ethanol-treated and non-ethanol-treated conditions, re-streaked them to purity, and performed full-length 16S ribosomal RNA gene sequencing to enable taxonomic characterization. Unique taxa were then archived as frozen stocks for future phenotypic analysis.

**Extended Data Figure 3:**
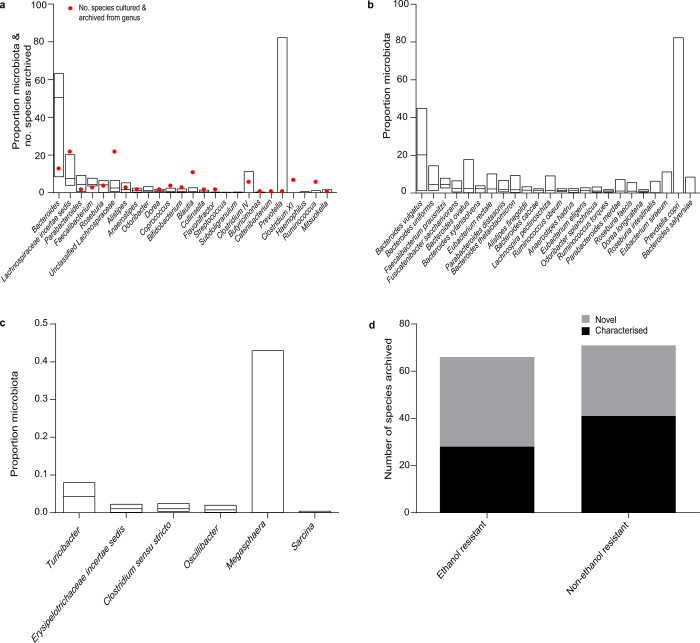
Archiving of bacterial diversity and novelty through anaerobic culturing. **a**, **b**, Representative species from 21 of the 25 most abundant bacterial genera (**a**) and 23 of the 24 most abundant species (**b**) were isolated and archived (abundance was determined by metagenomic sequencing and based on average relative abundance across the six donors (*n* = 6)). This represents 96% of the average relative abundance at the genus level and 90% of the average relative abundance at the species level across the six donors. A red dot in **a** indicates the number of species archived from each genus. *Lachnospiraceae incertae sedis*, unclassified *Lachnospiraceae*, *Clostridium* IV and *Clostridium* XI are not strict genera and represent currently unclassified species. *Odoribacter splanchnicus* in **b** was the only species not archived. **c**, Lowly represented intestinal microbiota members were also cultured. At least one representative species from each of the genera presented were cultured. Median and range is presented for the above with taxa ranked by median value. **d**, The number of bacterial species cultured in this study. At least 40% from each category were previously unknown.

In total, we archived bacteria representing 96% of the bacterial abundance at the genus level and 90% of the bacterial abundance at the species level based on average relative abundance across the six donors (Extended Data Fig. 3a, b). Even genera that were present at low average relative abundance (<0.1%) were isolated (Extended Data Fig. 3c). Overall, we archived 137 distinct bacterial species including 45 candidate novel species (Fig. 1c, Extended Data Fig. 3d and Supplementary Table 1), and isolates representing 20 candidate novel genera and 2 candidate novel families. Our collection contains 90 species from the Human Microbiome Project’s ‘most wanted’ list of previously uncultured and unsequenced microbes^16^ (Supplementary Table 1). Thus, our broad-range YCFA-based culturing approach led to massive bacterial discovery, and challenges the notion that the majority of the intestinal microbiota is unculturable.

**Extended Data Figure 4:**
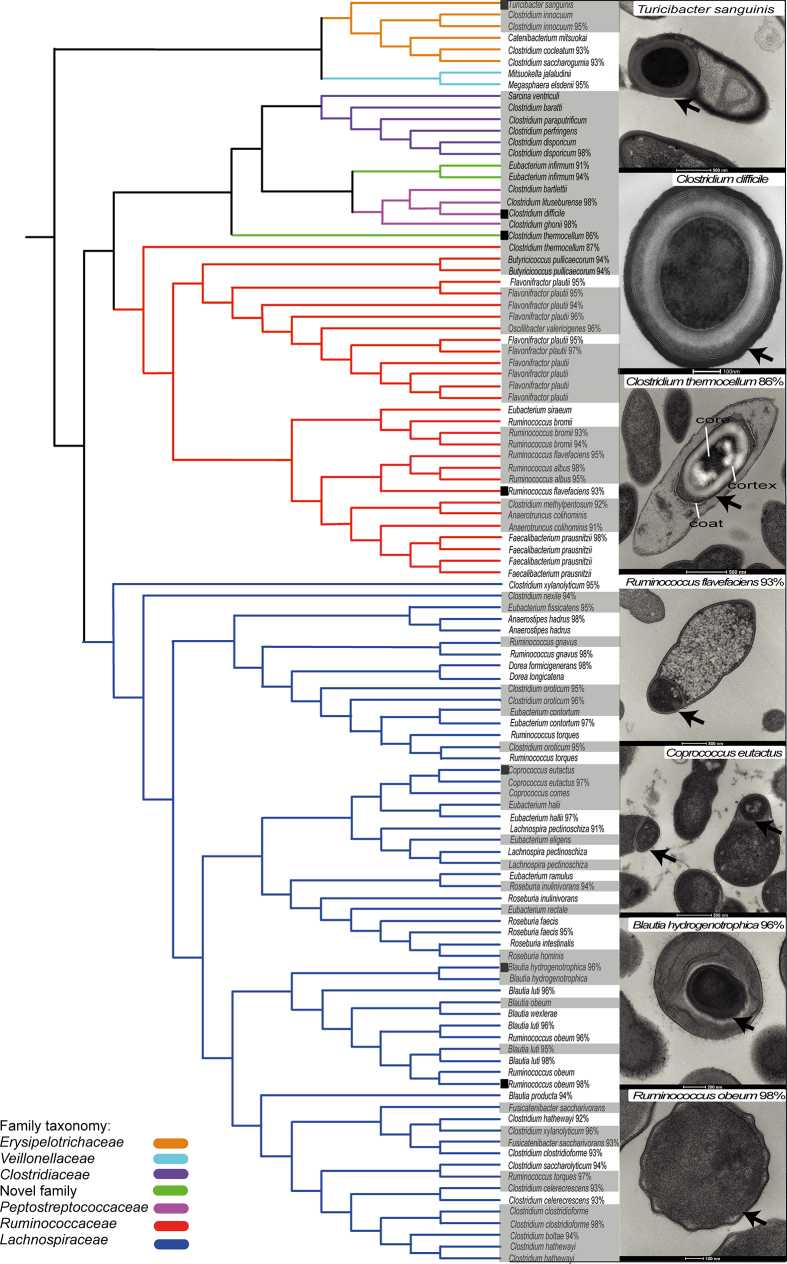
Phylogeny of intestinal spore-forming bacteria. Full length 16S rRNA gene phylogeny illustrating the taxonomic relationship of ethanol-resistant bacteria within the Firmicutes cultured from the donor faecal samples. Branch colours indicate distinct families. Shaded text indicates species cultured from an ethanol-treated faecal sample and unshaded text indicates species cultured from a non-ethanol-treated faecal sample. Percentage values represent closest identity to a characterized species. Transmission electron micrographs (TEMs) of spore ultrastructures for a phylogenetically diverse selection of cultured bacteria are shown with an arrow in images and include a candidate novel family with 86% identity to the 16S rRNA gene sequence from *Clostridium thermocellum*. Typical spore structures are defined and illustrated in the same image. TEMs are ordered according to boxes next to the species name. Scale bars are shown at the bottom of each image. *C. difficile* is included for context. Bacteria displaying an ethanol-resistant phenotype represent species previously classified as non-spore-formers (*Turicibacter sanguinis*^38^ and closely related candidate novel species), species closely related to non-spore-formers (*Roseburia intestinalis*^39^ and *Oscillibacter valericigenes*^40^ and closely related candidate novel species) or species suspected of forming spores but which, to our knowledge, have never been demonstrated to do so until now (*Eubacterium eligens*, *Eubacterium rectale*, *Coprococcus comes*^41^ and related candidate novel species).

**Extended Data Figure 5:**
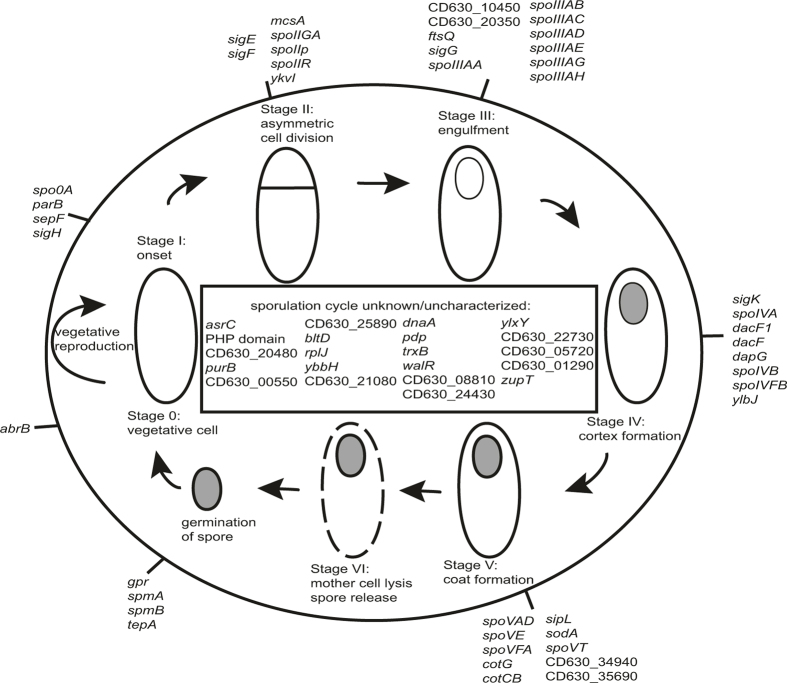
Genomic signature of sporulation within the human intestinal microbiome. A genomic signature for identifying spore-forming bacterial species contains sporulation- and germination-associated genes and genes not previously associated with sporulation. Characterized sporulation genes are on the outer circle, genes not associated with a specific sporulation cycle or uncharacterized genes are in the inside rectangle. *C. difficile* strain 630 gene names are used when possible, otherwise locus tag identifiers are shown. *Bacillus subtilis* gene names are used when no *C. difficile* homologue is available. The signature is enriched with known sporulation-associated genes from stages I–V of the spore formation and germination cycles (significant at *q* < 3.0 × 10^−37^, Fisher’s exact test). Genes associated with regulation are present with at least 10 genes coding for regulatory or DNA-binding roles (*q* < 1.4 × 10^−5^, Fisher’s exact test). Genes not previously associated with sporulation are also present and these have putative roles as heat shock, membrane-associated proteins and DNA-polymerase-associated proteins.

**Extended Data Figure 6:**
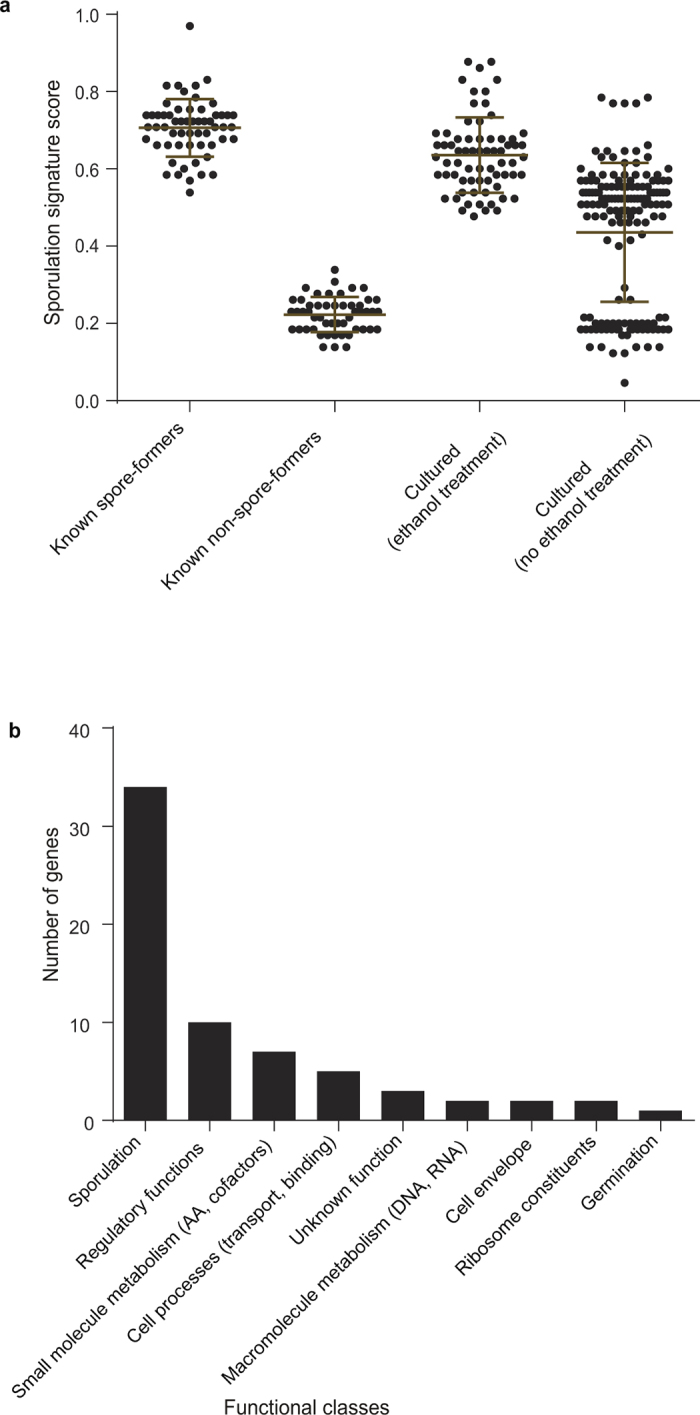
Validation and characterization of the sporulation signature. **a**, The signature accurately distinguishes spore-forming and non-spore-forming bacteria cultured from this study and from across different environments (known spore-formers *n* = 57, known non-spore-formers *n* = 50, cultured after ethanol treatment *n* = 69, cultured after no ethanol treatment *n* = 149). Refer to Supplementary Table 1 for signature scores of the bacteria tested. Mean ± s.d. **b**, Assignment of functional classes to the signature reveals a wide range of functional processes with sporulation- and regulation-associated genes dominating.

We isolated and purified bacteria representing 66 distinct ethanol-resistant species that are distributed across 5 known families and 2 newly identified candidate families (Extended Data Fig. 3d and Extended Data Fig. 4). The identification of these new and unexpected spore-formers highlights the broad taxonomic distribution of this phenotype among the enteric species of the Firmicutes. To define the conserved genetic pathways underlying sporulation and germination within the intestinal microbiota, we sequenced, assembled and annotated the whole genomes of 234 archived ethanol-resistant and ethanol-sensitive bacteria. Previously, the gene markers used to identify spore-forming bacterial species have been based on underlying genetic assumptions^13,17,18^; here we applied an unbiased computational approach to define 66 conserved genes linked to an ethanol-resistance phenotype (Extended Data Fig. 5 and Supplementary Table 1). This gene set allows for the prediction of the sporulation capabilities of bacterial species isolated from diverse environments with a high degree of accuracy (Extended Data Fig. 6a and Supplementary Table 1) and consists of genes from a wide range of functional classes (Extended Data Fig. 6b and Supplementary Table 1).

**Figure 2:**
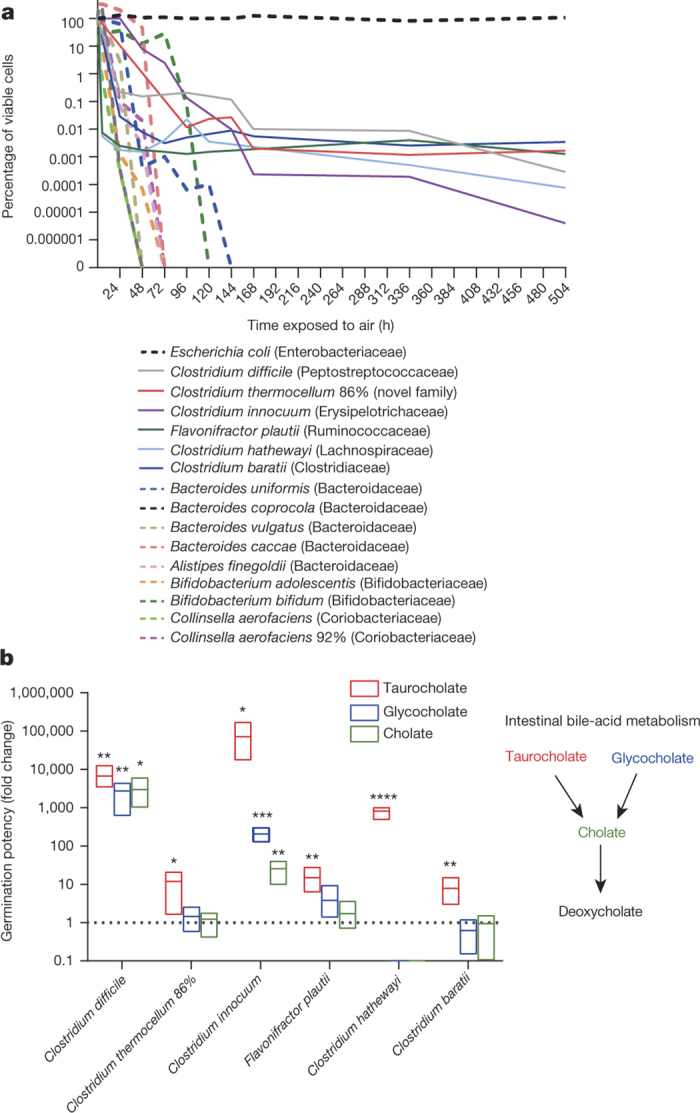
Phenotypic characterization of phylogenetically diverse intestinal spore-forming bacteria. **a**, Spore-formers are more aero-tolerant than non-spore-formers, which is expected to facilitate host-to-host transmission. Once exposed to oxygen, only 1% of the original inoculum of non-spore-forming bacteria (dashed lines) were viable after 96 h (4 days) and none were viable after 144 h (6 days). Spore-forming bacteria (solid lines) persist owing to spore formation. The experiment was stopped after 504 h (21 days). Taxonomic families of each species tested are shown in brackets (*n* = 3 biological replicates for each strain). **b**, Intestinal spore-formers respond to bile-acid germinants. The number of colony-forming units (c.f.u.) (representing germinated spores) present on plates in the presence of a particular germinant is expressed as a fold change with respect to the number of c.f.u. recovered on plates in the absence of a germinant. Spore-formers and non-spore-formers were subjected to ethanol shock before being plated (*n* = 6 biological replicates for each strain). Only spore-formers survived. A fold change of one (dashed line) would indicate that a germinant had no effect on the number of c.f.u. recovered. Schematic summarizes the cholate-derived bile acid metabolism in the mammalian intestine. Mean and range, Welch’s unpaired two-tailed *t*-test (*P < 0.05, **P < 0.01, ***P < 0.001, ****P < 0.0001). PowerPoint slide

**Extended Data Figure 7:**
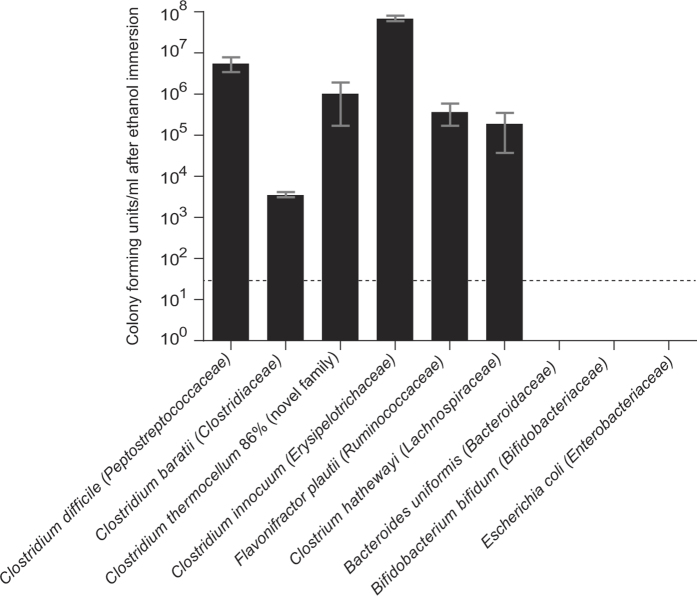
Spore-forming bacteria are more resilient than non-spore-forming bacteria to environmental stresses such as disinfectants. Pure bacterial cultures were immersed in ethanol for 4 h before being washed and inoculated onto YCFA growth medium with sodium taurocholate as a germinant. Only spore-forming bacteria survived. Taxonomic family names are shown in brackets. The dashed line indicates the culture detection limit of 50 c.f.u. ml^−1^. Mean ± s.d., *n* = 3 biological replicates for each species tested.

To test whether commensal spore formation facilitates long-term environmental survival, we exposed a phylogenetically diverse selection of commensal spore-forming and non-spore-forming bacteria and *C. difficile* to ambient oxygen for increasing periods of time. Under these conditions, non-spore-forming bacteria remained viable for 2–6 days (48–144 h) (Fig. 2a). In contrast, commensal spore-forming bacteria, *C. difficile* and the facultative anaerobe *Escherichia coli* were able to survive stably to the end of the experiment on day 21 (504 h). In addition, spore-forming commensals and *C. difficile*, but not non-spore-forming commensals, survived prolonged exposure to the common disinfectant ethanol (Extended Data Fig. 7). These results demonstrate that commensal spore-formers and *C. difficile* share a core set of sporulation genes that confer a highly resistant phenotype that is associated with environmental spread between humans.

**Extended Data Figure 8:**
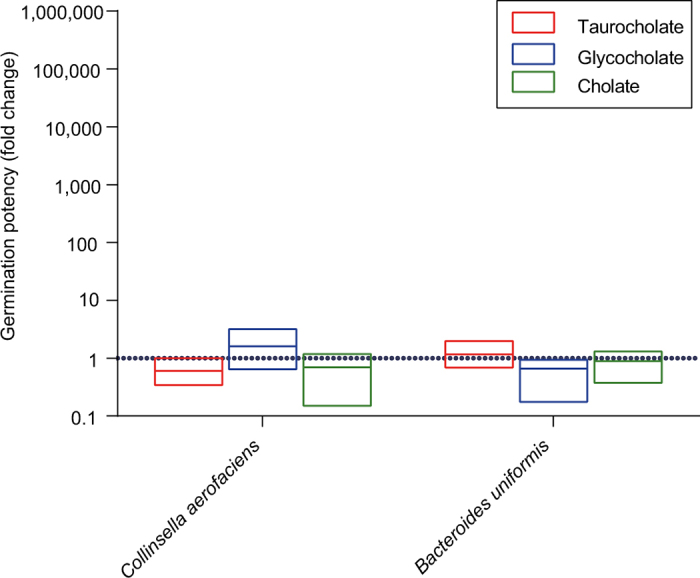
Growth response of non-spore-forming bacteria to intestinal germinants. The number of c.f.u. present on plates in the presence of a particular germinant expressed as a fold change with respect to the number of c.f.u. present on plates in the absence of a germinant. No ethanol shock treatment was performed beforehand. A fold change of one (dashed line) would indicate that a germinant had no effect on the number of c.f.u. recovered from the bacteria. There was no statistically significant difference based on an unpaired *t*-test of each germinant condition against the no germinant condition. Mean and range, *n* = 3 biological replicates for both species.

*C. difficile* spores have evolved mechanisms to resume metabolism and vegetative growth after intestinal colonization by germinating in response to digestive bile acids released into the small intestine from the gall bladder^9^. We exposed enteric spore-formers and non-spore-formers to common bile acids (taurocholate, glycocholate and cholate) to assess their response to germinants after ethanol-shock treatment (Fig. 2b). Taurocholate was a potent germinant for all spore-formers, increasing the culturability of spores from commensal bacteria by between 8- and 70,000-fold (*P* < 0.05 for all spore-formers tested), whereas the other cholate derivatives had varying efficacy in germinating commensal spore-formers (Fig. 2b). Taurocholate and the other bile acids had no impact on the culturability of non-spore-formers, demonstrating that the effect is specific to spore-formers (Extended Data Fig. 8). We propose that this bile-acid-triggered ‘colonizing germination’ mechanism serves as a conserved *in vivo* cue to promote colonization by intestinal spore-forming bacteria. Thus, a duality of purpose exists in the *modus operandi* of intestinal spore-forming bacteria; spore formation ensures their survival and transmission while germination in response to *in vivo* cues ensures their persistence in the human population.

**Figure 3:**
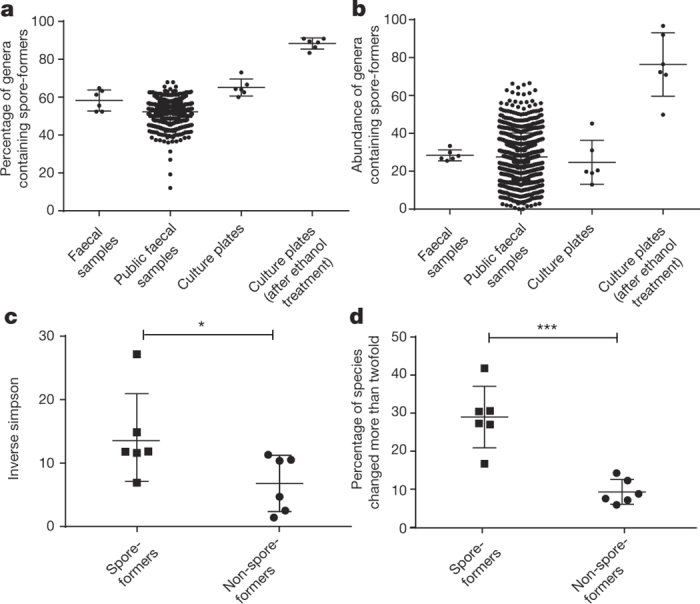
Extensive and dynamic sporulation capacity within the human intestinal microbiota. **a**, **b**, Using the genomic signature to interrogate public (*n* = 1,351) and complete faecal sample metagenomic data sets from this study (*n* = 6) reveals the proportion of spore-formers as a count of the total number of genera (**a**) and as total microbial abundance (**b**). **c**, **d**, Metagenomic sequencing of donor faecal samples (*n* = 6) 1 year later demonstrates that spore-forming bacteria are more diverse than non-spore-forming bacteria (**c**) and that a significantly increased proportion of species show twofold or greater change over the same time period (**d**). Mean ± standard deviation (s.d.), two-tailed paired *t*-test (*P < 0.05, ***P < 0.001). PowerPoint slide

**Extended Data Figure 9:**
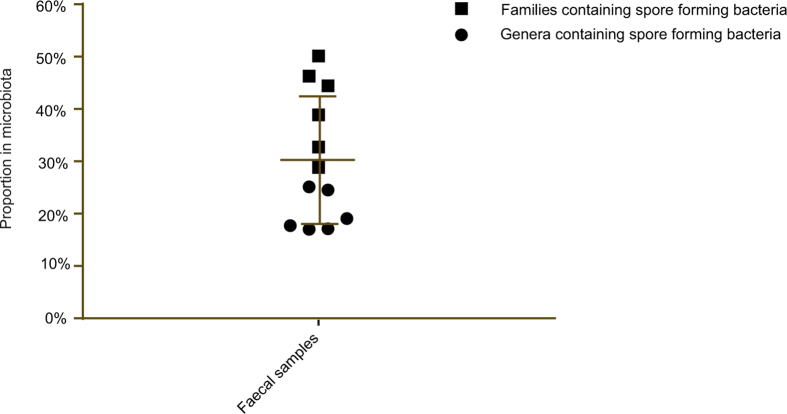
Validation of the estimation of the proportion of spore-formers in the intestinal microbiota. Full-length 16S rRNA gene amplicon sequencing was used to determine the taxonomic proportions of bacteria from the six donor faecal samples. Spore-forming bacteria were cultured from each donor and a taxonomic classification was assigned as described in the main text. The genus (circle) and family (square) taxonomic ranks were designated as the lower and upper limits for calculating the proportion of spore-formers at a taxonomic level. Specific genera and families were included if they contained a species that was cultured after ethanol shock treatment. Mean ± s.d.

We next sought to estimate the proportions of spore-forming bacteria within the intestinal microbiota. Interrogation of the metagenomic data sets with the spore gene signature predicted that, on average, 60% of the genera contained spore-forming bacteria (Fig. 3a). These genera represent 30% of the total intestinal microbiota (Fig. 3b). We independently validated these observations with 16S rRNA gene amplicon sequencing (Extended Data Fig. 9). Importantly, these proportions of spore-forming bacteria were also observed in 1,351 publicly available faecal metagenomic data sets generated from healthy individuals^19^ (Fig. 3a, b). We also found the same proportion of spore-formers (61.3%) within the ‘metagenomic species’ derived from 318 healthy individuals^4^.

While the intestinal microbiota is considered to be relatively stable over time^20^, evidence suggests that close contact of family members promotes sharing of Ruminococcaceae and Lachnospiraceae bacteria^21^, families that we describe as spore-formers (Extended Data Fig. 4). We noted that in our cohort, the spore-forming bacteria of the microbiota were significantly more diverse than the non-spore-forming bacteria (Fig. 3c). To test the dynamics of the spore-forming and non-spore-forming bacteria over time, we analysed the metagenomic profiles of faecal samples collected from the same healthy subjects one year after the original sampling. Interestingly, we noted a significantly increased variability in the proportion of spore-forming bacteria compared with non-spore-forming bacteria over this period. This suggests a higher species turnover or a greater shift in relative abundance in the spore-forming bacterial species (Fig. 3d). Taken together, our phenotypic and genome analyses demonstrate that the spore-forming and non-spore-forming bacteria represent major, distinct phenotypic components of our microbiota, each with unique colonization dynamics.

We show that spore formation is a widespread, although previously unappreciated function of the human intestinal microbiota, with important implications for microbiota transmission and inheritance. On the basis of the shared phylogeny and common evolutionary and phenotypic characteristics of sporulation and germination, we propose that the abundant commensal intestinal spore-formers identified here rely on the same transmission and colonization strategy as *C. difficile*^22^. In brief, environmental *C. difficile* spores are highly transmissible for long periods after they are shed, commonly transmit within a local environment but also have the potential to spread rapidly over long distances^23^. The transmission dynamics and geographical range of commensal spore-formers has yet to be determined, but we anticipate that this type of information will provide great insight into the heritability and the selective factors that shape the composition of the human intestinal microbiota.

Our workflow enables large-scale culturing, archiving, genome sequencing and phenotyping of novel bacteria from the human gut microbiota that were formerly considered to be unculturable. We have generated a sizable whole-genome-sequence data set that corresponds to 39% of the total number of intestinal bacterial genomes generated by the Human Microbiome Project. Our streamlined, single-medium approach, builds on the considerable efforts of others^24,25^ and unlocks the human intestinal microbiota for phenotypic characterization.

## Methods

### Culturing

Fresh faecal samples were obtained from six consenting healthy adult human donors (1 faecal sample per donor: minimum 0.5 g) and were placed in anaerobic conditions within 1 h of passing to preserve the viability of anaerobic bacteria. All sample processing and culturing took place under anaerobic conditions in a Whitley DG250 workstation at 37 °C. Culture media, PBS and all other materials that were used for culturing were placed in the anaerobic cabinet 24 h before use to reduce to anaerobic conditions. The faecal samples were divided in two. One part was homogenized in reduced PBS (0.1 g stool per ml PBS) and was serially diluted and plated directly onto YCFA^7^ agar supplemented with 0.002 g ml^−1^ each of glucose, maltose and cellobiose in large (13.5 cm diameter) Petri dishes. This sample was also subjected to metagenomic sequencing to profile the entire community. The other part was treated with an equal volume of 70% (v/v) ethanol for 4 h at room temperature under ambient aerobic conditions to kill vegetative cells. Then, the solid material was washed three times with PBS and it was eventually resuspended in PBS. Plating was performed as described earlier.

For the ethanol-treated samples, the medium was supplemented with 0.1% sodium taurocholate to stimulate spore germination. Colonies were picked 72 h after plating from Petri dishes of both ethanol-treated and non-ethanol-treated conditions harbouring non-confluent growth, (that is, plates on which the colonies were distinct and not touching). The colonies that were picked were re-streaked to confirm purity. No statistical methods were used to predetermine sample size. The experiments were not randomized. The investigators were not blinded to allocation during experiments and outcome assessment.

### Microbiota profiling and sequencing

Identification of each isolate was performed by PCR amplification of the full-length 16S rRNA gene (using 7F (5′-AGAGTTTGATYMTGGCTCAG-3′) forward primer and 1510R (5′-ACGGYTACCTTGTTACGACTT-3′) reverse primer followed by capillary sequencing. Full-length 16S rRNA gene sequence reads were aligned in the Ribosomal Database Project (RDP), manually curated in ARB^26^ and mothur^27^ was then used to classify reads to operational taxonomic units (OTUs). The R package seqinr version 3.1 was used to determine sequence similarity between OTUs and 98.7% was used as a species-level cut-off^28,29^. The full-length 16S rRNA gene sequence of each species-level OTU was compared to the RDP reference database to assign taxonomic designations to the genus level^30^ and a BLASTn search defined either a characterized or candidate novel species^31^.

Comparisons with the Human Microbiome Project (HMP) were carried out using 97% sequence similarity of the 16S rRNA gene sequence from the cultured bacteria to define a species because only partial 16S rRNA gene sequences were available. HMP data regarding the most wanted taxa and the completed sequencing projects were downloaded from http://hmpdacc.org/most_wanted/#data and http://hmpdacc.org/HMRGD/, respectively.

Genomic DNA was extracted from at least one representative of each unique OTU using a phenol-chloroform-based DNA isolation procedure. DNA was sequenced on the Illumina HiSeq platform generating read lengths of 100 bp and these were assembled and annotated for further analysis. DNA was extracted directly from each faecal sample for whole-community metagenomic and 16S rRNA gene amplicon sequencing using the MP Biomedical FastDNA SPIN Kit for soil. To enable comparisons with the complete community samples, non-confluent cultures were scraped from agar plates 72 h after inoculation with the initial faecal sample and DNA was extracted from this community using the same DNA isolation process. 16S rRNA gene amplicon libraries were made by PCR amplification of variable regions 1 and 2 of the 16S rRNA gene using the Q5 High-Fidelity Polymerase Kit supplied by New England Biolabs. Primers 27F AATGATACGGCGACCACCGAGATCTACAC (first part, Illumina adaptor) TATGGTAATT (second part, forward primer pad) CC (third part, forward primer linker) AGMGTTYGATYMTGGCTCAG (fourth part, forward primer) and 338R CAAGCAGAAGACGGCATACGAGAT (first part, reverse complement of 3′ Illumina adaptor) ACGAGACTGATT (second part, golay barcode) AGTCAGTCAG (third part, reverse primer pad) AA (fourth part, reverse primer linker) GCTGCCTCCCGTAGGAGT (fifth part, reverse primer) were used. Four PCR amplification reactions per sample were carried out; products were pooled and combined in equimolar amounts for sequencing using the Illumina MiSeq platform, generating 150 bp reads.

### Microbiota analysis

A maximum likelihood phylogeny of the culture-derived bacteria was generated from the aligned RDP sequence using FastTree version 2.1.3 (ref. 32) with the following settings: a generalized time-reversible (GTR) model of nucleotide substitution and CAT approximation of the variation in rates across sites with 20 rate categories. The ethanol-resistant phylogeny was derived directly from the entire culture phylogeny. All phylogenetic trees were edited in ITOL^33^.

Analysis of the partial 16S rRNA gene sequence generated from the 16S rRNA gene amplicon libraries was carried out using the mothur MiSeq SOP^34^ on 29 August 2014, generating 7,549 OTUs across all samples. A sequence similarity threshold of 97% was used to define an OTU.

Metagenomic sequence reads were analysed using the Kraken^35^ taxonomic sequence classification approach based on a custom database comprising complete, high-quality reference bacterial, DNA viral and archaeal genomes in addition to the genomes sequenced in this research. Resulting classified reads were log_2_ transformed and standardized by total abundance. Metagenomic samples were compared at the genus and species levels by relative abundance and at the genetic level by alignment using the bowtie2 algorithm^36^ to the appropriate gene catalogue. Sequences were considered present where an average of twofold coverage was achieved across the length of the considered sequence. A cut-off of 100 unique reads was applied to determine metagenomic species detection. Where appropriate, Spearman’s rank correlation coefficient was applied for correlation analysis. Inverse Simpson’s diversity index was calculated from Kraken output in R version 3.2.1 using the vegan: Community Ecology Package version 2.3-0.

### Gene sporulation signature

Heuristic based bidirectional best hit analysis was performed to identify 21,342 conserved genes within the 694,300 genes annotated across the 234 sequenced genomes. Support vector, machine-based, contrast set association mining was applied to identify the optimal, weighted gene signature consisting of 66 genes. Species classification was performed using BLAST-based gene detection with percentage detection weighted by gene signature contribution and scaled to generate a total score between 0 and 1. Scores greater than 0.5 were considered true spore-formers based on comparison to known spore-formers. Signature-based abundance was assessed against 1,351 publically available metagenomic data sets from healthy individuals^19^ after taxonomic assignment using the Kraken database. Genera were considered spore-formers when all known species within that genus had a spore forming score greater than 0.5.

### Transmission electron microscopy

Spore images were generated using transmission electron microscopy (TEM) as previously described^37^. Bacterial isolates for imaging were prepared by streaking pure cultures from frozen glycerol stocks and confirming purity by full-length 16S rRNA gene sequencing after one round of sub-culture to obtain visible and isolated single colonies. TEM images were prepared from culture plates 72 h after inoculation. The number of spore bodies visible in the TEM images was expressed as a percentage of the number of vegetative cells present and this ranged from 1% for *Ruminococcus flavefaciens*_93% to 4% for *Turicibacter sanguinis.*

### Oxygen sensitivity assay

Pure cultures were grown overnight in YCFA broth under anaerobic culture conditions as described earlier and the cultures were spotted in a dilution series onto YCFA agar containing 0.1% sodium taurocholate. Plates were incubated under ambient (aerobic) conditions at room temperature for specified time periods before being returned to the anaerobic cabinet. Colony-forming units (c.f.u.) were counted 72 h later. Cultures that were incubated anaerobically, and which were therefore not exposed to oxygen, acted as controls. Prior to the assay, all species were subjected to ethanol shock and were cultured anaerobically to determine their ability to sporulate. The viability of the oxygen-exposed cultures was expressed as a percentage of the viability of the anaerobic control cultures.

### Germination response to intestinal bile acids assay

Pure cultures were grown overnight in YCFA broth under anaerobic conditions and were then washed by repeatedly centrifuging to a pellet and re-suspending in PBS. Vegetative cells were killed using an ethanol shock treatment as previously described and the cultures were then serially diluted and plated on YCFA agar with and without 0.1% intestinal bile salts (taurocholate, cholate and glycocholate). Colony-forming units (c.f.u.) were counted 72 h later and the fold change of the number of c.f.u. present on plates in the presence of a particular germinant with respect to the number of c.f.u. present on plates in the absence of a germinant was calculated. The limit of detection (200 c.f.u. ml^−1^) was used for the number of c.f.u. recovered from *Clostridium hathewayi* plated without any germinants to allow a fold-change calculation. The experiment to determine the response of non-spore-formers to germinants was carried out similarly, except that vegetative cells were not treated with ethanol but rather were serially diluted and plated directly after washing.

## Supplementary information

Supplementary Tables This file contains Supplementary Table 1. (XLSX 129 kb)

## Data Availability

European Nucleotide Archive
ERP012217 Assembled and annotated genome sequence data have been deposited in the European Nucleotide Archive under accession number ERP012217. Bacterial isolates have been deposited at the Leibniz Institute DSMZ-German Collection of Microorganims and Cell Cultures (http://www.dsmz.de), the CCUG-Culture Collection, University of Gothenburg, Sweden (http://www.ccug.se), the Belgian Co-ordinated Collection of Micro-organisms hosted by the Laboratory of Microbiology (BCCM/LMG) at Ghent University (http://bccm.belspo.be/) and at the Japan Collection of Microorganisms (JCM; http://jcm.brc.riken.jp/en/). Isolate accession numbers are listed in Supplementary Table 1. Any isolates without accession numbers are available upon request.

## PowerPoint slides

PowerPoint slide for Fig. 1
PowerPoint slide for Fig. 2
PowerPoint slide for Fig. 3
