# Supplementary material for: Culturing of ‘unculturable’ human microbiota reveals novel taxa and extensive sporulation
Source: Nature. 2016 May 4;533(7604):543–6. doi: 10.1038/nature17645 (PMC4890681; doi:10.1038/nature17645)
Supplement: Supplementary file 4 — PowerPoint slide for Fig. 3 [file 41586_2016_BFnature17645_MOESM37_ESM.ppt]

## Slide 1
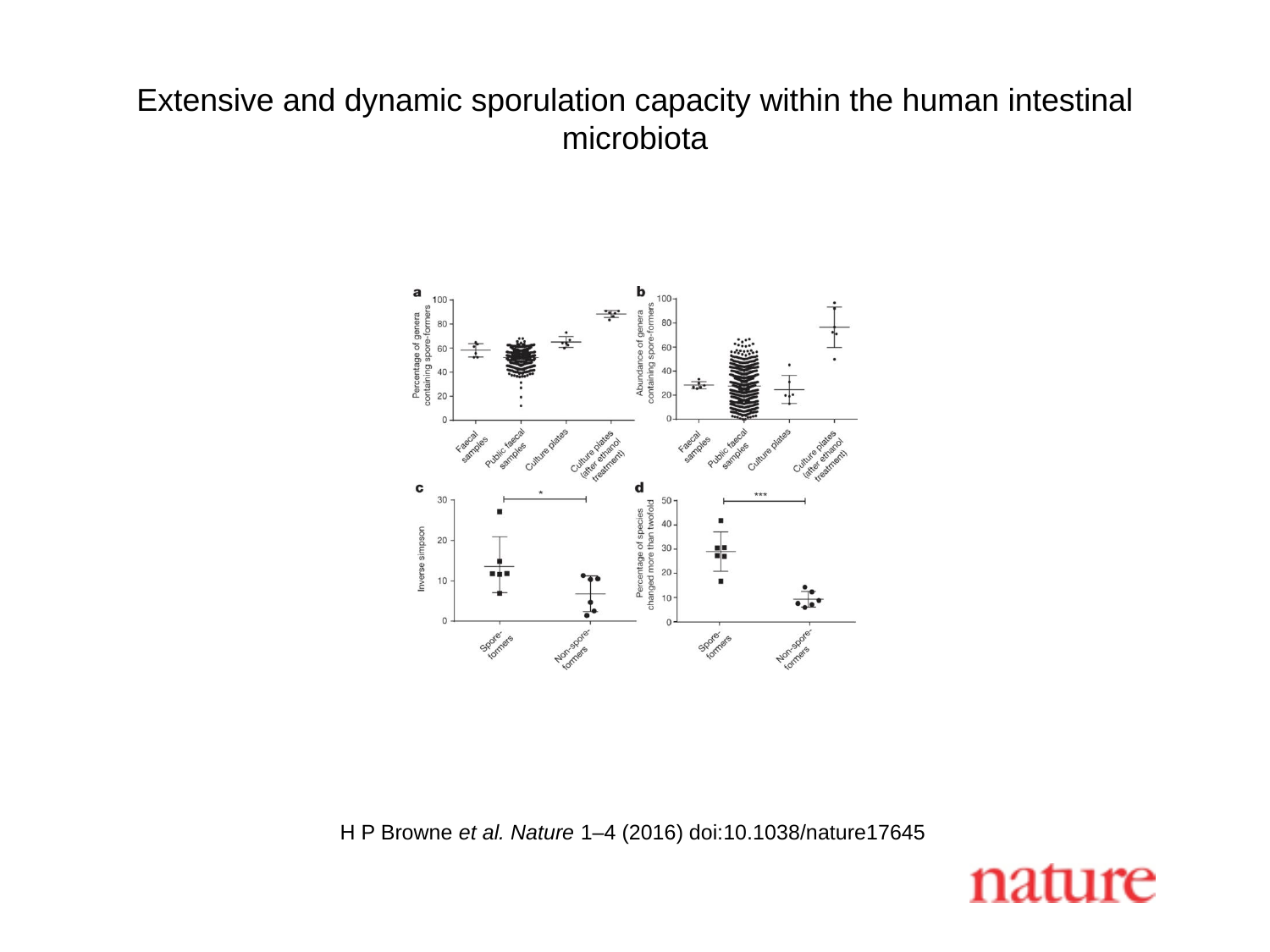

# Extensive and dynamic sporulation capacity within the human intestinal microbiota
H P Browne et al. Nature 1–4 (2016) doi:10.1038/nature17645
